# Hearing Loss and Blood Coagulation Disorders: A Review

**DOI:** 10.3390/hematolrep15030043

**Published:** 2023-07-04

**Authors:** Virginia Corazzi, Andrea Migliorelli, Chiara Bianchini, Stefano Pelucchi, Andrea Ciorba

**Affiliations:** ENT and Audiology Unit, Department of Neurosciences and Rehabilitation, University Hospital of Ferrara, 44124 Ferrara, Italy

**Keywords:** sensorineural hearing loss, sudden hearing loss, inner ear disease, blood coagulation disorders, clotting, bleeding

## Abstract

A relationship between microvascular disorders and sensorineural hearing loss (SNHL) has been widely proposed. The vascular hypothesis, theorized for the onset of sudden SNHL (SSNHL), is among the most acknowledged: a localized acute cochlear damage, of ischemic or haemorrhagic nature, could be considered a causative factor of SSNHL. The aim of this review is to assess (i) the effect on hearing in patients affected by blood coagulation disorders (prothrombotic or haemorrhagic) and (ii) the possible etiopathogenetic mechanisms of the related hearing loss. A PRISMA-compliant review was performed. Medline, Embase, and Cinahl databases were searched from inception to 31 January 2023, and a total of 14 studies have been included in the review. The available data suggest that it is possible to consider clotting disorders as a potential condition at risk for sensorineural hearing loss; in particular, coagulation tests and eventually the assessment of genetic and acquired prothrombotic factors should be recommended in patients with SSNHL. Also, an audiological evaluation should be recommended for patients with blood coagulation disorders presenting cochlear symptoms, especially in those suffering from clotting diseases.

## 1. Introduction

The vascularization of the inner ear is provided by a terminal capillary bed originating from the labyrinthine artery [1]. Given the absence of collateral vessels and the high oxygen-dependent metabolic activity of the inner ear cells, this sensorial organ is characterized by a high sensitivity to blood flow variations. Furthermore, blood flow does not pulsate towards the peripheral capillaries, where instead the circulation is passively regulated by systemic pressure variation and metabolic factors. This anatomic and functional configuration could clearly explain the extreme susceptibility of the inner ear to vascular accidents and peripheral vasomotion disorders, also in the presence of minimal and/or fleeting changes in partial oxygen tension [2]. In fact, there is great evidence, in the literature, of the relationship between microvascular disorders and sensorineural hearing loss (SNHL), which is defined as a loss of hearing function caused by the death of cochlear hair cells and/or neurons of the spiral ganglion and/or of the central auditory pathway. Microvascular disorders can irreversibly affect the inner ear, causing SNHL [3]. Furthermore, alterations in (i) blood viscosity, (ii) red cell deformability, and (iii) platelet aggregation have been hypothesized to be responsible for localized acute ischemic events in the inner ear [4,5]. Among the possible causes of sudden sensorineural hearing loss (SSNHL) [6], the vascular hypothesis (ischemic or haemorrhagic) is among the most acknowledged, as micro-thromboembolism or micro-haemorrhage of the internal auditory artery are considered possible causes of acute cochlear damage [7,8,9]. For this reason, the link between hearing loss and hypercoagulable states and bleeding diathesis has been investigated already.

The aim of this review is to assess (i) the effect on hearing in patients affected by blood coagulation disorders (both clotting and bleeding disorders) and (ii) the possible etiopathogenetic mechanisms of the related hearing loss.

## 2. Materials and Methods

The PubMed, Embase, and Cinahl databases were searched from inception to 31 January 2023. The research was performed according to Preferred Reporting Items for Systemic reviews and Meta-Analyses (PRISMA) criteria [10]; it was carried out independently and restricted to papers in English and involving humans (Table 1). The search strategy was performed according to the following MEDLINE and medical subjected hearing (MeSH) terms: Hearing Loss [MeSH], Hearing Loss, Sudden [MeSH], Labyrinth Disease [MeSH], Blood Coagulation Disorders [MeSH]. Exclusion criteria were (i) studies published in non-English languages; (ii) studies containing aggregated or duplicated data from previously published works; and (iii) syndromes and conditions associated with hearing loss (e.g., noise exposure). Full-text articles were obtained in cases where the title, abstract, or keywords suggested that the study might be eligible for this review.

Initially, the total number of papers identified was 76; other papers (6) were also identified from references in the published literature when all authors agreed about the reliability and importance of these manuscripts, for 82 papers in total. A total of 19 papers were selected by reading titles and abstracts. After reading the full texts, 14 papers were suitable for this review according to all authors.

## 3. Results

A total of 14 papers were included in this study; among these, 9 were case reports. Clotting disorders were investigated in 11 studies and bleeding disorders in 3 papers. A summary of the included studies is presented in Table 2.

### 3.1. Clotting Disorders

#### 3.1.1. Factor V Mutation

The V Leiden G1691A mutation was found to be significantly more frequent in a group of 100 patients with SSNHL than in 200 healthy controls (*p* = 0.001) [11], and in a group of 56 patients with SSNHL than in 95 controls with normal hearing levels (*p* = 0.02) [12]. Furthermore, a significantly increased frequency of multiple allelic mutations was found in SSNHL patients compared to controls (*p* = 0.0001) [11]. Differently, this mutation was not found to be more frequent in a group of 48 patients affected by SSNHL compared to 86 healthy subjects in the prospective cohort study by Cadoni et al. [13]. Finally, in a case–control study involving a group of 368 patients with deep-vein thrombosis and 395 non-thrombotic controls, the factor V Leiden G1691A mutation was not significantly correlated to SSNHL [14].

Three case reports were found in the literature. Lovato et al. [15] reported the onset of a unilateral SSNHL with a pure-tone average (average of the pure-tone thresholds at 0.5, 1, 2, and 4 kHz) of 33.8 dB in a 41-year-old woman presenting a factor V heterozygous state. Intravenous betamethasone disodium phosphate 4 mg and 250 mL of saline solution with mannitol 10% daily were administered over 11 days. A deep-vein thrombosis in the lower extremity and pulmonary embolism were subsequently diagnosed and an oral anticoagulant therapy was prescribed. The 2-month follow-up pure-tone audiometry showed complete hearing recovery. Two case reports described 48- and 42-year-old women, respectively, with a factor V heterozygous mutation presenting with cerebral sinus venous thrombosis and unilateral moderate SSNHL [16,17]. In both cases, after intravenous heparin therapy then switched to oral anticoagulants, complete hearing loss recovery was reported, even if audiometric data were not shown for the follow-up.

#### 3.1.2. Prothrombin Mutation

The prothrombin G20210A mutation was found to be more frequent in 100 patients with SSNHL than in controls (200 healthy subjects), with statistical significance (*p* = 0.001) [11]. Furthermore, a significantly increased frequency of multiple allelic mutations was found in SSNHL patients compared to controls (*p* = 0.0001) [11]. In two retrospective case–control studies, involving a group of 368 patients with deep-vein thrombosis vs. 395 non-thrombotic patients [14] and a group of 118 patients with SSNHL vs. 352 healthy subjects [18], respectively, the prothrombin G20210A mutation was considered a strong independent risk factor for SSNHL (*p* < 0.0001 and *p* = 0.0091, respectively). This finding was not confirmed by other studies: neither in a group of 56 patients with SSNHL compared to 95 control healthy subjects [12] nor in a group of 48 patients with SSNHL compared to 86 healthy subjects [13].

#### 3.1.3. Protein S Deficiency

A significant relation between SSNHL and abnormal levels of protein S was not found, neither in a group of 48 patients with SSNHL compared to 86 healthy controls [13], nor in 368 patients with deep-vein thrombosis compared to 395 non-thrombotic subjects [14].

According to the literature, there is only one case reporting [19] on a 34-year-old woman with protein S deficiency presenting sequential bilateral pantonal profound SSNHL. The patient was also affected by a small atrial septal defect and multiple acute cerebellar and cerebral microinfarcts. Rivaroxaban was administered (5 mg per day) together with 1 g per day of intravenous methylprednisolone. Partial hearing loss recovery was observed during the audiological follow-up.

#### 3.1.4. Protein C Deficiency

No significant association between SSNHL and abnormal levels of protein C was found in a group of 48 patients with SSNHL compared to 86 healthy subjects [13], or in 368 patients with deep-vein thrombosis compared to 395 non-thrombotic subjects [14].

#### 3.1.5. Activated Protein C Resistance (APCR)

No significant correlation between SSNHL and abnormal levels of activated protein C resistance (APCR) was found in a group of 48 patients with SSNHL compared to 86 healthy controls [13], or in 368 patients with deep-vein thrombosis compared to 395 non-thrombotic subjects [14].

In the case reported by Crassard et al., an increased APCR was described in a 42-year-old woman with a heterozygous mutation of factor V presenting with cerebral sinus venous thrombosis and unilateral moderate SSNHL [16]. Complete hearing loss recovery was observed in 6 months after intravenous heparin therapy therefore she was switched to an oral anticoagulant; nonetheless, audiometric data were not shown for the follow-up.

#### 3.1.6. Antithrombin III Deficiency

In a cohort of 14 patients with SSNHL compared to 50 healthy subjects [20], Zajtchuk et al. described abnormal values of antithrombin III in four patients, but no statistical analysis was performed. No significant relationship between SSNHL and abnormal levels of antithrombin III was found in a group of 48 patients with SSNHL compared to 86 healthy controls [13].

In a case report, a 48-year-old woman presenting with sequential bilateral SSNHL was affected by antithrombin III deficiency; no hearing threshold improvement was observed after corticosteroid therapy [21].

### 3.2. Bleeding Disorders

#### 3.2.1. Haemophilia A

In a cohort of 14 patients affected by SSNHL compared to 50 healthy subjects [20], no significant alterations in factor VIII levels were observed.

In the available literature, there is only one case reported so far. Kashiwazaki et al. [22] described unilateral conductive hearing loss in a 46-year-old man due to the presence of a temporal bone haemophilic pseudotumor inducing stenosis of the auditory external canal. A hearing threshold improvement was reported after the pseudotumor was removed by petrosectomy.

#### 3.2.2. Thrombasthenia

Schlegelberger et al. [23] described a case of a 22-year-old woman affected by Glanzmann’s thrombasthenia and hearing loss, also presenting triphalangia of the thumbs; the authors supposed a probable autosomal recessive syndrome. Nonetheless, no information about the onset and the features of the hearing loss was reported.

#### 3.2.3. Immune Thrombocytopenia (ITP)

Only one case report described bilateral hearing loss in a 5-year-old boy presenting acute ITP purpura and bilateral hearing loss due to bilateral spontaneous hemotympanum [24]. Nonetheless, no hearing test was described.

## 4. Discussion

Hearing loss represents a possible extra-haematological manifestation in patients affected by blood coagulation disorders. Due to the different types of clotting and bleeding conditions, non-haematological complications, including hearing loss, may develop with variable occurrence and different severity. This review aims to evaluate the possible relationship between blood coagulation disorders, of ischemic or haemorrhagic nature, and hearing loss. Among the papers identified, the majority are represented by case reports. This fact could suggest that (i) the occurrence of hearing loss is uncommon in patients affected by blood coagulation disorders, and that (ii) a pathogenetic correlation between hypercoagulative and hypocoagulative states and hearing impairment is difficult to prove.

### 4.1. Clotting Disorders and the Risk of Developing SSNHL

Conditions characterized by blood hypercoagulability may also increase the risk of thromboembolic events in the inner ear. In fact, a significant association between SSNHL and the prothrombin G2021, the platelet GlyIIIaA1/A2, and factor V Leiden G1691A mutations has been reported [25].

Among the proposed pathogenic mechanisms of SSNHL, the vascular hypothesis is the most widely accepted [26]. The anoxia or hypoxia induced by a sudden interruption of the blood supply could be responsible for the onset of SSNHL, considering the high sensitivity of inner ear cells to partial oxygen tension variations [27].

Consistent with the results of the present review, the factor V Leiden and the prothrombin G20210A mutations, which represent the most common and the second-most common inherited thrombophilia, respectively [25], were found to have a significant correlation with SSNHL [11]. These mutations occur more frequently in patients with SSNHL (factor V mutation) [12] and can represent a strong independent risk factor for SSNHL (prothrombin G20210A mutation) [14,18]. These findings support the hypothesis that those with inherited prothrombophilic conditions are at risk of developing SSNHL [28]. However, to date, there is no direct evidence of the pathogenic mechanisms underlying the inner ear damage and, in particular, the relationship between prothrombotic risk factors and SSNHL is still debated [13,29]. Furthermore, according to the results of this review, no significant correlation between SSNHL and deficiencies of protein S, protein C, antithrombin III, and abnormal levels of APCR has been observed [13,14].

When a cerebral sinus venous thrombosis is diagnosed, such as in the case reports by Crassard et al. [16] and Gattringer et al. [17], the pathogenic mechanism of SNHL could consist of an elevated intracranial pressure hampering inner ear fluid homeostasis. The cochlear damage could be (i) indirect, due to the transmission of elevated intracranial pressure to the endolymph (secondary to venous drainage impairment or to micro-thrombosis), or (ii) direct, due to the compression of the vestibulocochlear nerve. Finally, the presence of an atrial septum defect, a condition associated with an increased risk of cryptogenic ischemic stroke, as in the case report by Park et al. [19], could eventually be responsible for an acute ischemic event involving the inner ear and, thus, SSNHL [30].

### 4.2. Bleeding Disorders and SSNHL: Not a Convincing Association

According to the present review, only three case reports met the inclusion criteria when considering bleeding disorders. This fact firstly suggests that conditions of hypocoagulability seem to be not related to hearing loss. Nonetheless, bleeding diathesis could foster haemorrhages in unusual sites. Audiological examination should be considered in the diagnostic workup of these patients, since even a simple sneeze or cough may be responsible for intratympanic bleeding in the case of sudden and rapidly increased middle ear pressure [24]. Furthermore, in extremely rare cases, possible stenosis of the auditory external canal could be due to a pseudotumor of the temporal bone, which represents an extraordinary complication of haemophilia, as in a reported case [22].

Eventually, inner ear haemorrhage has been linked to SSNHL [8,9]. As for autoimmune bleeding disorders, such as ITP, putative damage to inner ear vessels may be immune-mediated. The inflammation of the small blood vessels, due to immune depositions at vessel walls, can hamper blood flow, therefore causing cochlear damage. However, so far, there is no evidence in the literature of SNHL or SSNHL in patients affected by bleeding diathesis (such as ITP, haemophilia, thrombasthenia, etc.). The presence of intracochlear haemorrhage has been previously linked to leukaemia, sickle cell disease, systemic lupus erythematosus, endolymphatic sac tumours, Von Hippel–Lindau syndrome, and anticoagulation therapy, in addition to post-traumatic and post-surgical iatrogenic forms [31,32].

### 4.3. Hearing Threshold Recovery in SSNHL Patients and Blood Coagulation Disorders

The literature data suggest that hearing threshold recovery in cases of SSNHL due to ischemic and/or haemorrhagic events is very poor, especially when the loss of function is bilateral and/or profound [33]. Moreover, in patients with SSNHL presenting prothrombotic risk factors, there are no clear data about the efficacy of anticoagulant therapy on hearing recovery [6]. However, the results of the present review are not conclusive on this topic.

## 5. Limits of the Study

A major drawback of this study is the lack of reliable and significant data, as almost half of the included studies are case reports. However, this fact may reflect (i) the rarity of hearing loss occurrence in patients suffering from blood coagulation diseases, and that (ii) clotting and bleeding disorders have been poorly investigated as causes of hearing loss. Furthermore, the lack of evidence could also be due to the current difficulties in investigating inner ear disorders, since there is an absence of proper diagnostic tools for evaluating the status of cochlear cells, or the inner ear’s microcirculation, despite the advances in diagnostic techniques over recent decades.

## 6. Conclusions

According to the available data, clotting diseases could be considered a potential condition with a risk of hearing loss. For this reason, even if a routine comprehensive haematological screening could be too expensive to be performed in all patients affected by hearing loss, audiologists should at least investigate patients presenting with SSNHL through coagulation tests and eventually assess them for genetic and acquired prothrombotic factors (such as the factor V Leiden and prothrombin mutations). This could allow the identification of those at high risk of thrombotic events. At the same time, haematologists should pay attention to audiological symptoms, and a hearing assessment should be recommended for patients with clotting disorders.

## 7. Future Directions

To date, a specific and standardized approach for investigating the pathogenetic mechanisms occurring in the inner ear, and particularly to evaluate the onset of hearing loss in patients affected by hypercoagulative and hypocoagulative states, is not available yet. In fact, it is still difficult to investigate the cochlear cells’ status and the condition of the inner ear’s microcirculation with the available standard diagnostic approaches.

However, considering the advancements over the last decades, modern magnetic resonance imaging (MRI) cannot just rule out retrocochlear pathologies, but can also provide a more detailed study of the inner ear compartments. The advent of 3-Tesla MRI has provided new imaging support for the morphological study of the inner ear. New MRI protocols, such as the three-dimensional fluid-attenuated inversion recovery (3D-FLAIR) [34] and three-dimensional fast imaging employing steady-state acquisition (3D-FIESTA) [35], have been recently implemented in order to better evaluate specific inner ear conditions, such as labyrinthine haemorrhage. In addition, quantitative synthetic MRI has been reported to better detect high signal intensity in the affected inner ear compared to the 3D-FLAIR sequences [36]. MRI sequences with contrast enhancement evaluation and 4 h delayed acquisitions demonstrated higher precision in defining the site and extension of the inner ear lesions, especially for fluid compartments [37]. An MRI-based volumetric analysis of inner ear fluids has been also recently proposed in order to indirectly evaluate endolymphatic hydrops [38].

On the contrary, specific ischemic events within the inner ear seem to be more difficult to detect, especially if temporary, by the available radiologic techniques. Nonetheless, new insights could arise from the use of higher magnetic fields (i.e., 7-Tesla and 9-Tesla—to date only employed in experimental situations), or by performing earlier MRI sequences, within 24–48 h, from the onset of hearing loss.

Implementing new diagnostic tools could improve diagnostic accuracy, allowing not just the detection of labyrinthine abnormalities but also, possibly, the identification of damaged blood–labyrinth barrier, thus providing important advantages in terms of prognostic information. It is likely that, in the near future, the use of new MRI protocols could provide more reliable data about the possible etiopathogenetic mechanisms of inner ear disorders.

## Figures and Tables

**Table 1:** Literature evaluation and selection, according to PRISMA criteria.

Total number of articles obtained by PubMed, Embase, and Cinahl search	76
Other papers from references in the published literature	6
Total number of papers identified	82
Papers excluded ^1^	63
Articles assessed for eligibility	19
Papers excluded ^2^	5
Total number of papers finally identified	14

^1^ Exclusion criteria were (i) studies published in non-English languages; (ii) studies containing aggregated or duplicated data from previously published works; (iii) syndromes and conditions associated with hearing loss (e.g., noise exposure). ^2^ Exclusion criteria were (i) other topics; (ii) no extractable data.

**Table 2:** Studies included in the present review.

Study, Year	Study Design	Participants				Control Group (N)	Blood Coagulation Disorders	Results
		N	Diagnosis	Age (Mean ± SD)	% Male			
Capaccio et al., 2007 [11]	Prospective cohort study	100	SSNHL	48.12 ± 14.6	56	200 healthy subjects	Factor V Leiden G1691A mutation; prothrombin G20210A mutation	Factor V Leiden G1691A and prothrombin G20210A mutations were found to be more frequent in patients with SSNHL than in controls (*p* = 0.001). A significantly major frequency of multiple allelic mutations was found in SSNHL patients compared to controls (*p* = 0.0001).
Görür et al., 2005 [12]	Cohort study	56	SSNHL	42.6 ± 18.2 (10–87 range)	48.2	95 healthy subjects	Factor V Leiden G1691A mutation; prothrombin G20210A mutation	Factor V Leiden mutation was found to be more frequent in patients with SSNHL than in controls (*p* = 0.02). No significant difference was found between the groups in regard to prothrombin G20210A mutation (*p* = 0.58). Factor V Leiden and prothrombin mutations were heterozygous in all subjects.
Cadoni et al., 2006 [13]	Prospective cohort study	48	SSNHL	♂ 46 (21–74 range); ♀ 50 (22–75 range)	41.7	48 healthy subjects	Factor V Leiden G1691A mutation; prothrombin G20210A mutation; APCR; protein S deficiency; protein C deficiency; antithrombin III deficiency	No significant association was found between SSNHL and factor V G1691A mutation or prothrombin G20210A mutation or APCR or abnormal levels of protein S, protein C, and antithrombin III.
Mercier et al., 1999 [14]	Retrospective case–control study	368	Deep-vein thrombosis	41 (17–72 range)	27.4	395 non-thrombotic subjects	Factor V Leiden G1691A mutation; prothrombin G20210A mutation; protein S deficiency; protein C deficiency; antithrombin III deficiency	Prothrombin G20210A mutation was found to be an independent risk factor for unilateral SSNHL (*p* < 0.0001). No significant association was found between SSNHL and factor V Leiden G1691A mutation or abnormal levels of protein S, protein C, and antithrombin III.
Lovato et al., 2004 [15]	Case report	1	Unilateral SSNHL	41	0	N/A	Factor V Leiden G1691A mutation	Unilateral SSNHL onset with a pure-tone average (average of the pure-tone thresholds at 0.5, 1, 2, and 4 kHz) of 33.8 dB in a woman presenting a factor V heterozygous state. Intravenous betamethasone disodium phosphate 4 mg and 250 mL of saline solution with mannitol 10% daily were administered over 11 days. A deep-vein thrombosis in the lower extremity and pulmonary embolism were subsequently diagnosed and an oral anticoagulant therapy was prescribed. The 2-month follow-up pure-tone audiometry showed complete hearing recovery.
Crassard et al., 1997 [16]	Case report	1	Unilateral SSNHL	48	0	N/A	Factor V Leiden G1691A mutation; APCR	Unilateral moderate SSNHL onset presenting with cerebral sinus venous thrombosis in a woman with factor V heterozygous state and increased APCR. After intravenous heparin therapy then switched to oral anticoagulants, complete hearing loss recovery was reported in 6 months (audiometric data not shown).
Gattringer et al., 2012 [17]	Case report	1	Unilateral SSNHL	42	0	N/A	Factor V Leiden G1691A mutation	Unilateral moderate SSNHL onset presenting with cerebral sinus venous thrombosis in a woman with a factor V heterozygous state. After intravenous heparin therapy then switched to oral anticoagulants, complete hearing loss recovery was reported (audiometric data not shown).
Patscheke et al., 2001 [18]	Case–control study	118	SSNHL	45.5	57.6	352 healthy subjects	Prothrombin G20210A mutation	In a group of patients in which the first episode of SSNHL occurred before the age of 40, a statistically significant major frequency of prothrombin G20210A mutation was observed compared to control subjects and a 16-fold increased risk for SSNHL in carriers of the mutation was found (OR = 16, 95% CI 1.95 to 202; *p* = 0.0091).
Park et al., 2001 [19]	Case report	1	Bilateral SSNHL	34	0	N/A	Protein S deficiency	Sequential bilateral pantonal profound SSNHL onset in a woman with protein S deficiency. The patient also presented a small atrial septal defect and multiple acute cerebellar and cerebral microinfarcts. Rivaroxaban was administered (5 mg per day) together with 1 g per day of intravenous methylprednisolone. Partial hearing loss recovery was observed, during the audiological follow-up.
Zajtchuk et al., 1979 [20]	Case–control study	14	SSNHL	N/A	N/A	50 healthy subjects	Antithrombin III deficiency; factor VIII	Abnormal values of antithrombin III and factor VIII were reported in 4 and 1 patients, respectively. No statistical analysis was performed due to the small sample.
Gold et al., 1993 [21]	Case report	1	Bilateral SSNHL	48	0	N/A	Antithrombin III deficiency	Sequential bilateral SSNHL onset in a woman with antithrombin III deficiency; no hearing threshold improvement was observed after the corticosteroid therapy.
Kashiwazaki et al., 2012 [22]	Case report	1	Unilateral conductive hearing loss	46	100	N/A	Haemophilia A	Unilateral conductive hearing loss onset in a man due to the presence of temporal bone haemophilic pseudotumor inducing stenosis of the auditory external canal. An improvement in the hearing threshold was reported after removal of the pseudotumor by petrosectomy.
Schlegelberger et al., 1986 [23]	Case report	1	Hearing loss	22	0	N/A	Thrombasthenia	Presence of hearing loss, triphalangia of thumbs, and Glanzmann’s thrombasthenia, suggesting a probable autosomal recessive syndrome. Information about the onset and the features of the hearing loss was not reported.
Fisgin et al., 2009 [24]	Case report	1	Bilateral hearing loss	5	100	N/A	ITP	Bilateral hearing loss onset in a boy with acute ITP purpura. A bilateral spontaneous hemotympanum was diagnosed. No hearing test was reported.

Abbreviation: SSNHL, sudden sensorineural hearing loss; APCR, activated protein C resistance; N/A, not available; ITP, immune thrombocytopenia.

## Data Availability

Not applicable.
